# Anisotropy in spinodal-like dynamics of unknown water at ice V–water interface

**DOI:** 10.1038/s41598-023-43295-4

**Published:** 2023-10-11

**Authors:** Hiromasa Niinomi, Tomoya Yamazaki, Hiroki Nada, Tetsuya Hama, Akira Kouchi, Tomoya Oshikiri, Masaru Nakagawa, Yuki Kimura

**Affiliations:** 1 Institute of Multidisciplinary Research for Advanced Materials, Tohoku University, 2-1-1 Katahira, Aoba-ku, Sendai, Miyagi 980-8577 Japan; 2 Institute of Low Temperature Science, Hokkaido University, Kita-19, Nishi-8, Kita-ku, Sapporo, Hokkaido 060-0819 Japan; 3 Graduate School of Engineering, Tottori University, 4-101 Koyama-Cho Minami, Tottori, Tottori 680-8552 Japan; 4 Komaba Institute for Science, The University of Tokyo, 3-8-1 Komaba, Meguro, Tokyo 153-8902 Japan; 5 Research Institute for Electronic Science, Hokkaido University, Kita-21, Nishi-10, Kita-ku, Sapporo, Hokkaido 001-0021 Japan

**Keywords:** Structure of solids and liquids, Surfaces, interfaces and thin films, Wetting, Fluids, Liquid crystals, Phase transitions and critical phenomena, Cryospheric science

## Abstract

Experimentally demonstrating the existence of waters with local structures unlike that of common water is critical for understanding both the origin of the mysterious properties of water and liquid polymorphism in single component liquids. At the interfaces between water and ices I_h_, III, and VI grown/melted under pressure, we previously discovered low- and high-density unknown waters, that are immiscible with the surrounding water. Here, we show, by in-situ optical microscopy, that an unknown water appears at the ice V–water interface via spinodal-like dynamics. The dewetting dynamics of the unknown water indicate that its characteristic velocity is ~ 90 m/s. The time evolution of the characteristic length of the spinodal-like undulation suggests that the dynamics may be described by a common model for spinodal decomposition of an immiscible liquid mixture. Spinodal-like dewetting dynamics of the unknown water transiently showed anisotropy, implying the property of a liquid crystal.

## Introduction

Water is so abundant on the Earth that its properties govern various phenomena in nature. Thus, it is crucial to understand the origin of the mysterious properties of water unlike those of other liquids^1^, such as its density reaching its maximal value at 4 °C. A key for understanding the origin of the properties of water is experimental confirmation of the existence of waters with local structures that differ from that of common water. This importance stems from the mysterious properties of water explained by regarding water as a dynamical mixture of two types of local structures^2,3^ (i.e., disordered normal-liquid structure and the locally favored tetrahedral structure) or as the supercritical state of two kinds of liquids^4,5^ (i.e., a high-density liquid (HDL) and a low-density liquid (LDL)). Whether supercooled water macroscopically separates into a HDL and a LDL via liquid–liquid phase separation (LLPS) near the condition of thermodynamic singularity is a matter of debate^4,6,7^. Extensive efforts, involving both experiments and simulations, have been devoted to uncover the existence of such local structures from molecular-scale microscopic perspectives based on optical spectroscopy^8,9^, X-ray^10,11^, and neutron-scattering experiments^12,13^ in the supercritical regime, and molecular dynamics (MD) simulations^14^. These efforts have strongly supported the existence of waters with local structures different from that of water at the microscopic level. In addition, these experiments have suggested the existence of the Widom line which possibly emanates from the liquid–liquid critical point (LLCP) as a signature of macroscopic LLPS of water^10^.

**Figure 1:**
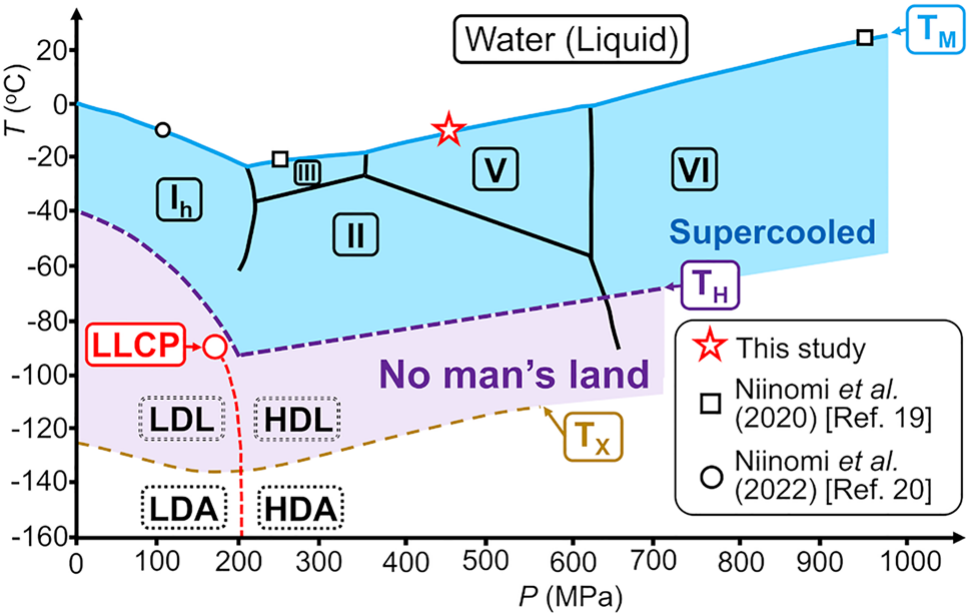
Phase relations of water, and the conditions for the in-situ observations. The red star indicates the conditions for observing the interface between water and ice V in the present study. The black squares indicate the conditions for observing the interface between water and high-pressure ices III and VI in a previous study^19^. The black circle indicates the condition for observing the interface between water and ice I_h_ in a previous study^20^. The solid blue line labeled by T_M_ shows the melting temperatures of the ices. The liquid water below T_M_ is metastable supercooled water. The region where supercooled water can exist is highlighted in blue. The purple dashed line labeled by T_H_ represents the temperature at which homogeneous nucleation inevitably hinders supercooling of water; the temperature region below T_H_ is therefore the so-called “no-man’s land”, highlighted in purple. The dashed yellow line labeled by T_X_ is the amorphous ice crystallization line. The red circle indicates the liquid–liquid critical point (LLCP). The red dashed line emanating from the LLCP is the expected first-order transition line between LDL (low-density amorphous ice, LDA) and HDL (high-density amorphous ice, HDA) above (below) T_x_. Stable, metastable, and predicted metastable phases are indicated by the rectangles drawn with black solid, dotted, and dotted double lines, respectively. The phase relations of this figure are adopted from Ref. ^21^ originally with modification. Reprinted with permission from Ref.^20^. Copyright 2022 American Chemical Society.

However, the macroscopic LLPS of water has never been observed because the hypothesized LLCP is predicted to lie in experimentally inaccessible region of conditions, known as “no man’s land”, where deep supercooling is hindered by instantaneous crystallization beyond an experimentally accessible timescale (Fig. 1)^10,11^. Therefore, experimentally demonstrating the macroscopic LLPS of water is difficult.

Moreover, experimental confirmation of the LLPSs of other single component liquids predicted to exhibit LLPS, such as silicon^15^, carbon^16^, hydrogen^17^, and nitrogen^18^ are also hindered by the similar experimental difficulties to achieve the thermodynamic conditions of LLCPs. These situations highlight the importance of the experimental detection of signatures leading to the macroscopic separation of water even under thermodynamic conditions that differ from the predicted LLCP.

Amid this situation, we have unexpectedly discovered, by simple *in-situ* optical microscopy, the existence of droplets and layers of unknown waters macroscopically separated from the surrounding bulk water at the nonequilibrium interfaces between water and ices grown/melted by applying pressure using a sapphire anvil cell^19,20^. The immiscibility of the unknown water with the bulk water strongly suggests the local structure of the unknown water differs from that of the bulk water. The existence of unknown waters has been confirmed at the interfaces of ice I_h_ and high-pressure ices III and VI under thermodynamic conditions in water–ice two-phase coexistence: (− 10 °C, 107 MPa), (− 20 °C, 248 MPa), and (25 °C, 954 MPa), respectively (Fig. 1). The densities of the unknown waters were suggested to be similar to those of ices supporting the unknown waters rather than the surrounding bulk water. These findings suggest that at least two kinds of low- and high-density unknown waters exist because the densities of ice I_h_ and high-pressure ices are lower and higher than that of bulk water, respectively. The separation of low- and high-density unknown waters from bulk waters does not necessarily correspond to the LLPS of water. Nevertheless, exploration of the properties, dynamics, and local structures of the unknown waters at the interfaces should provide insights not only into the possibility of macroscopic separation of waters with different local structures from water but also into liquid polymorphism in single component systems^22^ and the elementary processes of ice crystal growth from water beyond the frameworks of classical crystal growth theory^23^.

Here, we discovered that a high-density unknown water appears also at the interface between water and ice V by in-situ optical microscopy in a two-phase coexistence condition (− 10 °C, 443 MPa). The in-situ observations revealed that (1) the characteristic velocity of the unknown water is ~ 90 m/s, which corresponds to about two-fold of that of bulk water (~ 40 m/s)^24^, (2) the unknown water can appear via spinodal-like dynamics that may follow the predictions of a common model used to describe the dynamics of spinodal decomposition of a binary liquid mixture, and (3) the pattern of spinodal-like dewetting shows *anisotropy,* implying that the unknown water transiently exhibits the properties of a liquid crystal.

## Results and discussion

**Figure 2:**
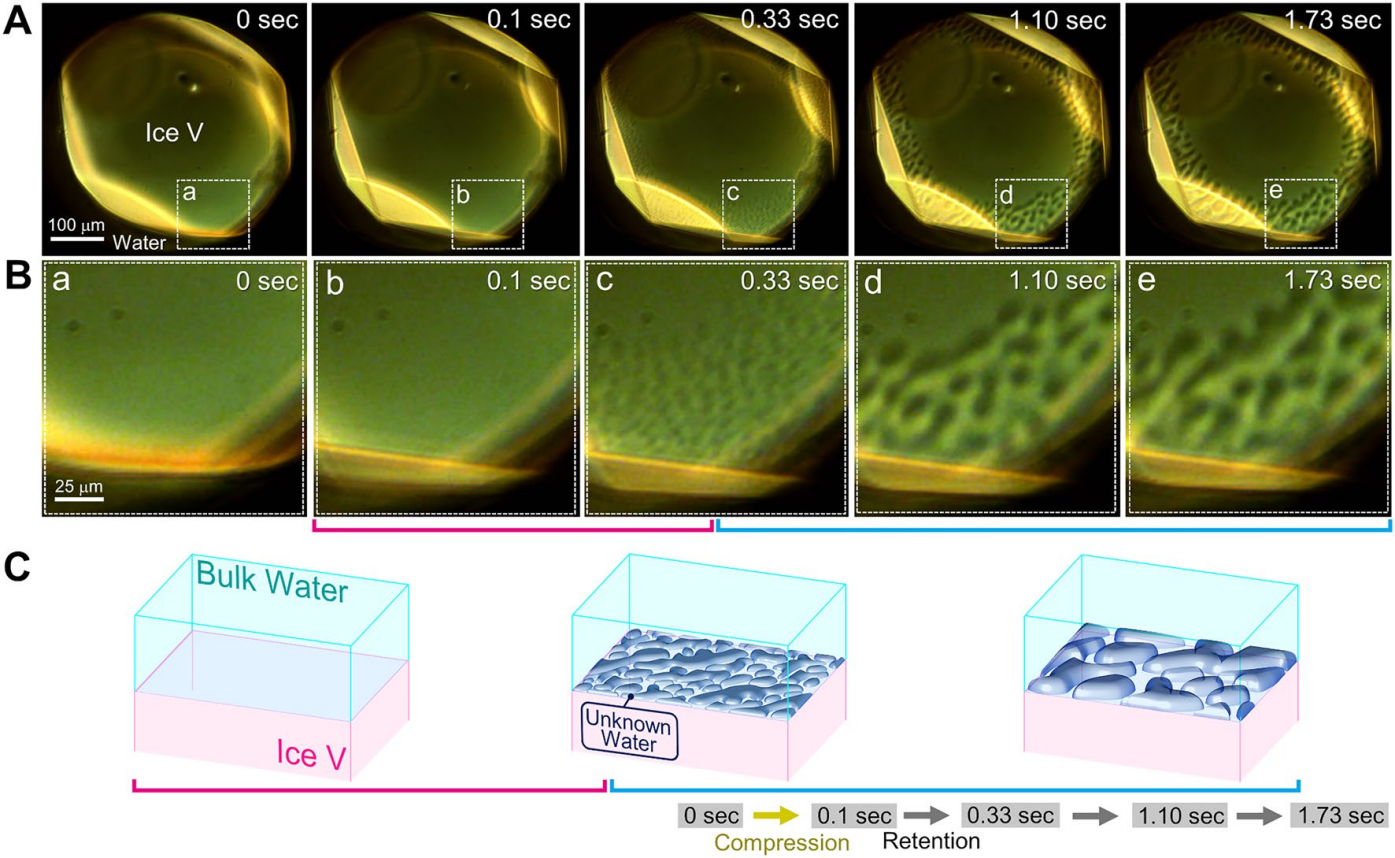
Time-lapse micrographs showing the appearance of unknown water through spinodal-like dynamics at the interface between water and ice V grown by compression. (**A**) Time-lapse micrographs captured by the in-situ observations, where the micrograph at 0 s shows the initial state before compression and the images collected at 0.10–1.73 s are time-lapse micrographs after compression. The applied pressure was retained during acquisition of the micrographs at 0.10–1.73 s in (**A**). (**B**) Magnified images of the region indicated by the white dashed squares denoted by a–e. (**C**) Schematics showing the time evolution of the morphology of the unknown water. The magenta and cyan solid lines indicate the corresponding schematic for the time evolution of the morphology shown in micrographs (**B**) 0.10–1.73 s underlined with magenta and cyan solid lines. The yellow and gray arrows in the right-hand bottom corner show the operations of compression and retention, respectively. See also SI Video S1.

Figure 2 shows time-lapse in-situ differential interference contrast micrographs of nonequilibrium interface between water and ice V (SI Video S1), which was grown by compression in a dynamic sapphire anvil cell (d-SAC) (See Method, Supplementary Information (SI) Figs. S1 and S2). Unknown water layers separated from the surrounding bulk water by clear interfaces exhibited a bicontinuous morphology when an effective overpressure^20,25^ of ~ 2.3 GPa was applied using a d-SAC to drive ice V crystal growth at the interface^20,26^. This overpressure is estimated to correspond to a thermodynamic driving force for crystallization of ~ 8.7 × 10^−21^ J^20^. The interface of the ice V exhibited a smooth morphology before the compression (0 s in Fig. 2A and B). Upon compression, numerous droplet-like domains with a few micrometers in size appeared at the interface of the ice V and bulk water within 0.2 s (0.1 and 0.33 s in Figs. 2A and B, respectively). The droplet-like domains grew over time and coalesced with each other, indicating fluidity of the unknown layer. In addition to the coalescence, the unknown water exhibited nucleation-and-growth-type dewetting dynamics from its thin layer (SI Text S1 and SI Video S2). This behavior also indicates fluidity of the unknown water. Analyses of the dewetting dynamics enabled us to estimate the characteristic velocity (i.e., the ratio between interfacial tension and viscosity) of the unknown water as a measure of fluidity (SI Text S1).

It is known that that characteristic velocity has a relationship with the spreading velocity of hole of the dewetting and dynamic wetting angle. The characteristic velocity was estimated to be ~ 90 m/s by spreading velocity measurements and dynamic wetting angle estimation with interferometry. It should be mentioned that the characteristic velocity of unknown water at the interface between ice III and water was previously estimated to be ~ 100 m/s from a relationship characteristic velocity and static wetting angle under the assumption that experimentally estimated dynamics wetting angle is equal to static wetting angle^20^. This value of characteristic velocity turns to be ~ 40 m/s if we apply the relationship of characteristic velocity and dynamic wetting angle used in this study. This suggests the characteristic velocity of the unknown water on ice V is 2.25-fold larger than that on ice III. In addition, the wetting angle of the unknown water was estimated to be ~ 0.56° by interferometric measurement. This angle suggests that the density of the unknown water is possibly higher than that of the surrounding bulk water (SI Text S3).

**Figure 3:**
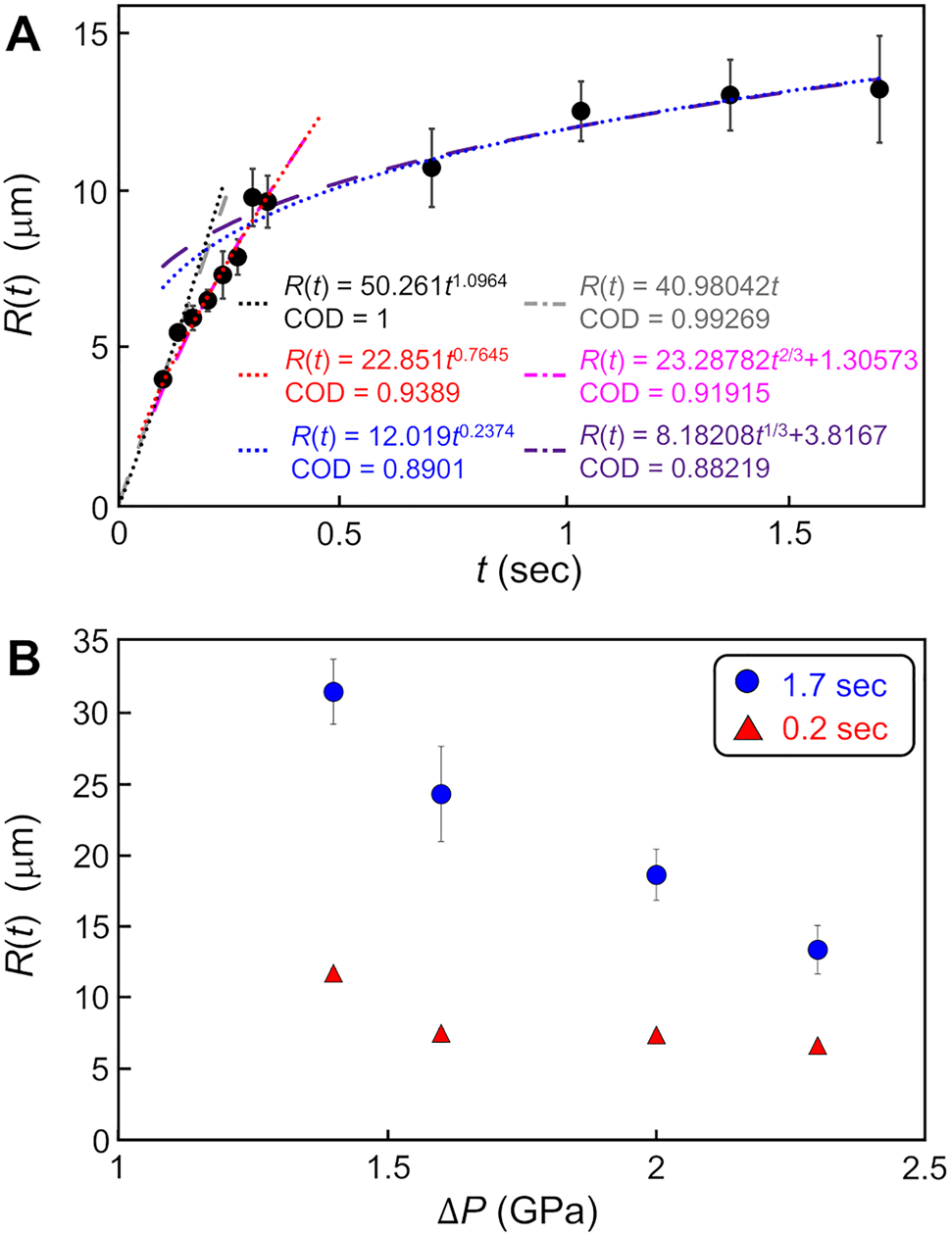
Time evolution of the characteristic length of the bicontinuous morphology in the spinodal-like generation dynamics of the unknown water, and the dependency of the characteristic length on the effective overpressure at 0.2 s and 1.7 s. (**A**) Time evolution of the characteristic length of the spinodal-like dynamics of the unknown water. The black dots indicate plots obtained by in-situ observation. Dotted lines are the curves fitted by the least-squares method using power-law functions with respect to time *t*. Black, red, and blue fitted curves corresponds to stages (1), (2), and (3) classified by the change of the power-law exponent *n*. COD is the coefficient of determination. The gray, magenta, and purple dashed lines are the curves fitted by the power-law functions with fixed exponents *n* = 1, 2/3 and 1/3 for the viscous regime, inertial regime and the late stage of spinodal LLPS described by model H, respectively. (**B**) The dependency of the characteristic length on the effective overpressure, which is proportional to the thermodynamic driving force. The plots of red triangles and blue circles indicate the dependency at 0.2 and 1.7 s after the compression, which correspond to stages (1) and (3), respectively.

The coalescence of the droplets was found to evolve to the bicontinuous morphology in 0.33–1.73 s (Figs. 2A and B, SI Video S1). Such a bicontinuous morphology is known to be observed in LLPS of a binary immiscible liquid mixture via spinodal decomposition and spinodal thin film instability^27^. To explore similarity between the dynamics of the unknown water with bicontinuous morphology and that generally observed in spinodal LLPS of a binary liquid mixture, we compared the time evolution of characteristic length, *R*(*t*), of the bicontinuous morphology of the unknown water and that predicted by existing theories which describes spinodal LLPS of a binary liquid mixture. Figure 3A shows plots of the characteristic length as a function of on time (SI Text S4). The time evolution was well fitted by a power-law time dependency *t*^*n*^. The fitting analysis suggested the existence of three stages classified according to the change of the exponent *n*: (1) *n* ≈ 1.10, (2) *n* ≈ 0.76, and (3) *n* ≈ 0.24. The intercept in the fitting equation for stage (2) and (3) was neglected to reduce arbitrariness arising from superfluous fitting parameter, although the intercept should be imposed by the continuity of the growth of the characteristic length. On the other hand, the dynamics of spinodal LLPS is known to be described by so-called model H^28^, which can take fluid dynamics into consideration in the dynamics of spinodal decomposition by introducing an additional convective term into the Cahn–Hilliard equation^29^ to couple with the Navier–Stokes equation. For a binary liquid mixture with a symmetric and critical composition, model H predicts a power-law time dependency of the characteristic length based on dynamical scaling law and several regime changes in the course of the progress of the spinodal decomposition^30^. The change of the regime results in a change of the power-law exponent *n*. The correspondences between the regimes and their time dependencies are as follows:

1 $$R\left( t \right) \propto \left\{ {\begin{array}{*{20}l} {t^{1/3} } \hfill & {\left( {{\text{diffusive}}} \right)} \hfill \\ t \hfill & {\left( {{\text{viscous}}} \right)} \hfill \\ {t^{2/3} } \hfill & {\left( {{\text{inertial}}} \right)} \hfill \\ \end{array} } \right.$$

Immediately after spinodal quenching, namely, in the early stage, molecular diffusion is the dominant process driving transportation because interface between the two liquids is unclear. This diffusive regime yields the power-law exponent *n* = 1/3. After the interface forms between the two liquids, the contribution of hydrodynamics governed by the Navier–Stokes equation becomes relevant and the dynamics depends on whether the surface tension is balanced by viscous and inertial forces. This balance causes blanching of the regime into the viscous and inertial regimes. At the initial stage after the hydrodynamics start to contribute, the transport is dominantly governed by the viscous term of the Navier–Stokes equation rather than by the inertial term. This viscous regime yields the exponent *n* = 1. The relationship between the contributions of the viscous and inertial terms can be inverted with time because of the hydrodynamic motion, leading to crossover from the viscous regime to the inertial regime. This inertial regime yields the exponent *n* = 2/3. Notably, this change from the viscous regime to the inertial regime is peculiar to model H. Interestingly, the exponents of the stages (1) and (2) in our observations (*n* ≈ 1.10 and 0.76, respectively) are similar to those predicted by model H for the viscous and inertial regimes (*n* = 1 and 2/3, respectively), although a regime corresponding to the diffusive regime was not apparent (Fig. 3A). This lack of a diffusive regime might be attributable to the scale of the bicontinuous morphology being too small to be observed by optical microscopy. In addition, the exponent of stage (3) in our observation (*n* ≈ 0.24) is similar to that associated with the Ostwald ripening process in the late stage of phase separation (*n* = 1/3)^31^: The plots could be well fitted by power functions with the exponents predicted by theories of LLPS (Fig. 3A). The discrepancy between the exponents in our plots and those predicted by theories may be attributable to the difference in the dimensions of the systems: Theories and our observations correspond to three and quasi-two-dimensional systems, respectively. In addition, the dependency of the characteristic length on the thermodynamic driving force also showed a similarity to that predicted by the theory of spinodal decomposition. Figure 3B shows the dependency of the characteristic length on the effective overpressure, which is proportional to the thermodynamic driving force, during stages (1) and (3). The characteristic length tended to decrease with increasing thermodynamic driving force. On the other hand, the Cahn–Hilliard equation predicts that a spinodal wave with a specific wavenumber preferentially amplifies in the early stage of spinodal decomposition. The specific wavenumber, *β*_*m*_, is determined by the following equation^32^:

2 $$\beta_{m} = \left[ { - \frac{{\left( {G^{\prime\prime}_{0} + 2\eta^{2} Y_{{\left\langle {hkl} \right\rangle }} } \right)}}{4K}} \right]^{\frac{1}{2}}$$

where $G^{\prime\prime}_{0}$ is the second derivative of free energy with respect to the order parameter, $\eta$ is the lattice mismatch, $Y_{{\left\langle {hkl} \right\rangle }}$ is the elastic modulus for the direction $\left\langle {hkl} \right\rangle$, and *K* is the gradient energy. Parameter $G^{\prime\prime}_{0}$ corresponds to the thermodynamic driving force and it is always negative in the condition where spinodal decomposition occurs. Thus, the specific wavenumber increases with increasing thermodynamic driving force. Because characteristic length, which corresponds to the wavelength of the spinodal wave, is inversely proportional to wavenumber, Eq. (2) indicates that the characteristic length decreases with increasing thermodynamic driving force. Assuming that the unobservable characteristic length in the diffusive regime influences those in the regimes of the later stages, this tendency is similar to that of the bicontinuous morphology of the unknown water. These similarities suggest that the observed dynamics of the unknown water is spinodal-like. On the other hand, the spinodal-like dynamics of the unknown water may be understandable also by thin film instability because the bicontinuous morphology can be observed also in spinodal thin film instability, which occurs when the thickness of a liquid thin film is below the critical thickness^33^. The reason why the bicontinuous morphology can be observed in spinodal thin film instability is that the development of surface fluctuation in spinodal thin film instability can be described by a Chan-Hilliard-type equation mathematically analogous to Cahn–Hilliard equation. However, here we avoid discussing the possibility of spinodal thin film instability as the origin of the bicontinuous morphology in detail. This is because that (1) the possibility of the spinodal thin film instability is based on an uncertain assumption that the unknown water is a phase which can nucleate and form a thin film. (2) The characteristic length in spinodal thin film instability depends on thickness and viscosity of liquid thin film, making discussion complicated. (3) Time evolution of characteristic length of bicontinuous morphology in spinodal thin film instability is unclear. Although we cannot conclude whether the origin of the bicontinuous morphology is spinodal LLPS or spinodal thin film instability at present, it should be mentioned that both mechanisms imply that the unknown water may be a phase and that dynamics of the unknown water may be able to be described within the frameworks of existing spinodal theories.

**Figure 4:**
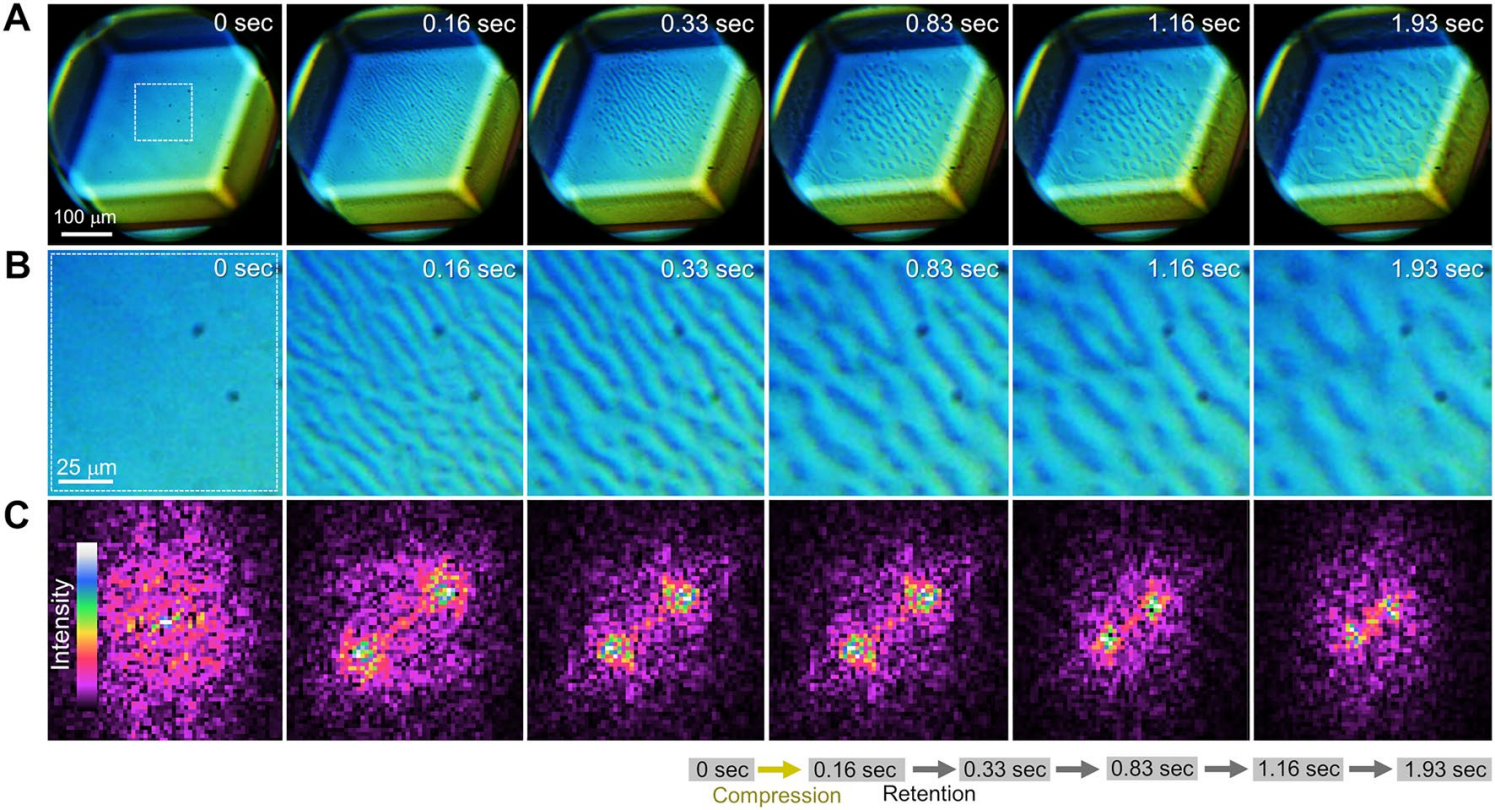
Time lapse micrographs showing anisotropy in spinodal-like dewetting dynamics of the unknown water at the interface between water and ice V grown by compression. (**A**) Time-lapse micrographs captured by the in-situ observations, where the micrograph at 0 s shows the initial state before compression and those at 0.16–1.93 s are time-lapse micrographs after compression. The applied pressure was retained at 0.16–1.93 s. (**B**) Magnified images of the region indicated by the white dashed square. (**C**) Fast Fourier transforms of the micrographs in (**B**). The yellow and gray arrows in the right-hand bottom corner show the operations of compression and retention, respectively. See also SI Video S3.

Notably, we found that *anisotropy* can be observed in spinodal-like dewetting dynamics of the unknown water. Figure 4 shows time-lapse micrographs of an *in-situ* observation of the $\left(0\overline{1 }0\right)$ face of ice V. The crystal face was estimated by the law of constant angle because the angles comprising the parallelogram face are almost identical to those comprising the $\left(0\overline{1 }0\right)$ face of the unit cell determined by X-ray diffraction analysis^34^. A spinodal-like wave elongated in the $\langle 101\rangle$ direction was observed within 0.16 s after compression corresponding to 2.3 GPa effective overpressure (Fig. 4A and B, SI Video S3). Fast Fourier transformation of the micrograph clearly showed anisotropy of the spinodal-like wave (Fig. 4A 0.16 s). The direction of elongation of the wave is always along the same crystallographic direction. This implies that the anisotropy originates from the crystallographic anisotropy of ice V. An anisotropic spinodal wave generally occurs during spinodal decomposition of solid phases described by the Cahn–Hilliard equation because of the contribution of the anisotropy to the elastic strain energy, lattice mismatch, interfacial energy, and diffusion coefficient, as partially shown by the corresponding terms in Eq. (2). However, the anisotropy is generally a characteristic peculiar to the solid phase. Anisotropy during spinodal dewetting of a liquid layer has scarcely been reported because liquids are generally isotropic. Such reports have been limited to systems of polymer thin films on a patterned or rubbed substrate^35^. The occurrence of the anisotropy requires that the unknown water anisotropically interacts with the ice V crystal surface at sufficiently long-range to cover the thickness of the unknown water layer, which is approximately 90 nm (SI Text S1). Such a long-range interaction is possibly an elastic interaction. However, if the unknown water is a general isotropic liquid, no elastic interaction should occur between the unknown water layer and the ice V. This discrepancy implies the possibility that the unknown water transiently exhibits the properties of a liquid crystal. This possibility is feasible because it is reasonable to recognize the unknown water as a transient intermediate state between liquid water and crystalline ice. In this context, it should be mentioned that some of hydrogen-bonded water molecules in crystal structure of ice V are known to display significant orientational order even though ice V is classified as a hydrogen-disordered phase of ice^36^. This anisotropy in the hydrogen-bonded water molecules with orientational order may transiently remain immediately after the formation of the unknown water. These considerations are relevant to the picture of the recently discussed crystal growth beyond classical crystal growth theory^37^, so-called nonclassical crystal growth^38^.

Whereas classical crystal growth theory excludes the existence of an intermediate state between the crystal phase and the mother phase, recent experimental studies related to nonclassical crystal growth from a solution have suggested that molecules in solution transiently change their state to a state thermodynamically more compatible with the crystalline state, such as a dense liquid droplet or an amorphous precursor, before the incorporation of molecules into the crystal. In addition, a recent simulation suggested that “preordering” of a melt at the interface plays an important role in crystal growth from a supercooling melt^23^. Especially, a recent study showed that plastic crystal intermediate layer forms at the interface between water and a growing ice VII crystal by molecular dynamics simulations^39^. The possibility that the unknown water transiently exhibits the property of a liquid crystal is consistent with the picture of nonclassical crystal growth. This suggests the possibility of nonclassical melt growth of ice^23,40^. On the other hand, the formation of structured interfacial water in nano-confinement has been suggested by combinations of measurements using surface force apparatus (SFA)^41,42^. It is known that a water confined between two hydrophilic surfaces below 1 nm shows viscosity 7 orders of magnitude larger than that of bulk water^42^. The existence of a structured interfacial water layer is the most accepted explanation for this phenomenon. Although it is not easy to directly compare the structured interfacial water and the unknown water because their systems are different in many points, e.g. pressure range, scale and so on, the existence of waters with the structures different from that of bulk water is a common characteristic. The structure of water under nanoconfinement is known to be strongly influenced by surface structures or properties of container. Thus, the structure of the unknown water may be the consequence of the influence by the surface structure of ice V crystal. Although these possibilities are not conclusive, they should be further explored in the future.

## Conclusions

Our in-situ optical microscopic observation revealed that unknown water separated from the surrounding bulk water appears at the interface between water and ice V grown by compression using a d-SAC. The in-situ observations revealed various dynamics of the unknown water, droplet formation, spinodal-like bicontinuous morphology formation, nucleation-and-growth type dewetting and spinodal-like dewetting. Analyses on the nucleation-and-growth-type dewetting dynamics enabled us to estimate the characteristic velocity of the unknown water to be ~ 90 m/s. The time evolution of the characteristic length of the bicontinuous morphology in the spinodal-like dynamics of the unknown water was found to show similarity to the dynamics predicted by a model used to describe spinodal decomposition in LLPS (i.e., model H). In addition, the dependency of the characteristic length on the thermodynamic driving force was found to be similar to that predicted by the Cahn–Hilliard equation. These similarities imply that the spinodal-like dynamics of the unknown water may be described by the frameworks of existing spinodal theories. Moreover, the in-situ observation showed *anisotropy* in the spinodal-like dewetting dynamics of the unknown water. This anisotropy implies that the unknown water can transiently exhibit the properties of a liquid crystal although this possibility is not conclusive. These findings related to the properties, dynamics, and local structure of the unknown water provide insights not only into the LLPS of water, which is key to understanding the origin of the mysterious properties of water, but also into elementary processes of crystal growth from a melt and liquid polymorphism in single component liquids.

## Methods

The experimental setup of dynamic sapphire anvil cell (d-SAC) was described also in the previous study^20,26^. Three piezo actuators (N 20/S 10, P-232-40; Piezosystem Jena GmbH, Jena, Germany) were embedded between stainless steel sheets supporting a pair of sapphire anvils in a symmetrical-type SAC (SEED; Syntek Co. Ltd, Yokohama, Japan). The d-SAC was also equipped with a manual pressure-adjustment system using a screw (SI Fig. S1). This design allowed dynamic and fine control of (de)pressurization by actuating the piezo elements with a piezo amplifier (E-662; PI, Auburn, MA, USA) connected to a function generator (33522B; Keysight, Santa Rosa, CA, USA) in addition to coarse adjustment using the pressure-adjusting screw. Ultrapure water produced by an ultrapure-water-producing apparatus (Simplicity UV; Merck Millipore, Burlington, MA, USA) fed with distilled water (Kyoei Seiyaku Co., Tokyo, Japan) was used as the mother liquid for crystallization of ice V. Ice V was crystallized by compressing the ultrapure water in its sample chamber in a low-temperature room maintained at − 10 °C. After crystallization of ice V polycrystals, the polycrystals were melted by decompression in the d-SAC; the pressure was controlled by using the coarse pressure adjustment to the pressure conditions for the coexistence of water and ice V (~ 443 MPa). Then, the crystals were repeatedly melted and grown by decompression and compression with the coarse adjuster so that eventually only a single crystal remained in the sample chamber. A water–ice V interface was produced by growing the single crystal so as not to completely crystallize the coexisting water in the sample chamber. After producing the interface, the ice V crystal was repeatedly grown and melted by pressurization/depressurization through fine adjustment by the piezo actuators. The piezo elements were actuated by applying square-wave voltages from the function generator. The frequencies and the peak-to-peak voltages for the square waves from the function generator were set to be 250 mHz and 1, 2, 3, 4, 5, 6 or 7 V_pp_, respectively. The voltage was applied to the piezo actuators after a ten-fold amplification by the amplifier. According to the analyses on the dependency of crystal growth rate of ice I_h_ on the applied voltage in the previous study^20^, the set voltages were estimated to result in the effective overpressure at the interface^25^ of about 0.33, 0.67, 0.98, 1.3, 1.6, 2.0 and 2.3 GPa, respectively. The analyses also showed that these effective overpressures correspond to the thermodynamic driving force for crystallization of 1.2 × 10^–21^, 2.5 × 10^–21^, 3.7 × 10^–21^, 4.9 × 10^–21^, 6.2 × 10^–21^, 7.2 × 10^–21^ and 8.7 × 10^–21^ J, respectively. The interface between the water and the ice V crystal repeatedly grown and melted in synchronization with the applied square wave was observed in-situ by bright-field microscopy, differential-interference phase-contrast microscopy, and Fizeau-type laser interferometric microscopy using an inverted optical microscope (IX71; Olympus Corp., Tokyo, Japan) set in the low-temperature room. The microscopic images were recorded in-situ by using a CCD camera (UI-3180CP-C-HQ Rev.2; IDS Image Development Systems GmbH, Obersulm, Germany). The bright-field and differential-interference phase-contrast microscopy was conducted by using a set of commercially available optical components (Olympus Corp.). The laser interferometric microscope was constructed by combining a He–Ne laser (05-LHR-211; CVI Melles Griot, Albuquerque, NM, USA; λ  = 632.8 nm) with the inverted optical microscope (SI Fig. S2). The laser beam emitted in the horizontal direction was first introduced into an objective lens (SLMPLN20X; Olympus) and then passed to a polarization beam splitter (PBS) to introduce the laser light into the optical path of the inverted optical microscope by reflecting it in the vertical direction. The light reflected by the PBS was next introduced through an objective lens (SLMPLN20X; Olympus) for sample observation; this lens collimated the introduced light. The ice V crystal was irradiated by the collimated laser beam, and the incident laser light and the light reflected by interfaces interfered with each other to produce interference fringes (SI Fig. S2).

### Supplementary Information

Supplementary Information 1.

Supplementary Video 1.

Supplementary Video 2.

Supplementary Video 3.

Supplementary Video 4.

Supplementary Video 5.

## Data Availability

The data is available from H.N. on e-mail request (hiromasa.niinomi.b2@tohoku.ac.jp).
