# Supplementary material for: Anisotropy in spinodal-like dynamics of unknown water at ice V–water interface
Source: Sci Rep. 2023 Oct 11;13:16227. doi: 10.1038/s41598-023-43295-4 (PMC10567706; doi:10.1038/s41598-023-43295-4)
Supplement: Supplementary file 1 — Supplementary Information 1. [file 41598_2023_43295_MOESM1_ESM.docx]

Supplementary Information for

Anisotropy in Spinodal-like Dynamics of

Unknown Water at Ice V–Water Interface

Hiromasa Niinomi*^†^, Tomoya Yamazaki^‡^, Hiroki Nada^§^, Tetsuya Hama^¶^, Akira Kouchi^‡^, Tomoya Oshikiri^†‖^, Masaru Nakagawa^†^, and Yuki Kimura^‡^

^†^ Institute of Multidisciplinary Research for Advanced Materials, Tohoku University, 2-1-1 Katahira, Aoba-ku, Sendai, Miyagi, 980-8577, Japan

^‡^ Institute of Low Temperature Science, Hokkaido University, Kita-19, Nishi-8, Kita-ku, Sapporo, Hokkaido, 060-0819, Japan

^§^ Graduate School of Engineering, Tottori University, 4-101 Koyama-Cho Minami, Tottori, Tottori, 680-8552, Japan

^¶^ Komaba Institute for Science, the University of Tokyo, 3-8-1 Komaba, Meguro, Tokyo 153-8902, Japan

‖Research Institute for Electronic Science, Hokkaido University, Kita-21, Nishi-10, Kita-ku, Sapporo, Hokkaido, 001-0021, Japan

Corresponding author: Hiromasa Niinomi

Email: [hiromasa.niinomi.b2@tohoku.ac.jp](mailto:hiromasa.niinomi.b2@tohoku.ac.jp)

**This PDF file includes:**

SI Texts S1 to S4……………………..................................................Page S2-S6

SI Figures S1 to S5…………………………………………………...Page S7-S11

Legends for SI Videos S1 to S5………………………………….......Page S12

SI References………………………………………………………....Page S13

**Other supplementary materials for this manuscript include the following:**

SI Videos S1 to S5

Supplementary Information Text S1

**Estimation of the characteristic velocity of the unknown water at the water-ice V interface.**

Analysis of the nucleation-and-growth-type dewetting dynamics of a thin layer of the unknown water permitted its characteristic velocity at the water-ice V interface to be determined (Figure S3). It is known that a liquid thin film on a substrate with a negative spreading coefficient spontaneously shows dewetting when the thickness of the thin film, *e*, falls below a critical thickness, *e*_c_. The dewetting of a liquid thin film often follows nucleation-and-growth-type dynamics, where a hole nucleated in the thin film at a certain location grows by spreading radially. In this growth process, the velocity at which the hole spreads (*V*_h_) can be expressed by the following equation if the relationship of dynamic wetting angle of the unknown water* _D_* << 1 rad and *e* << *e*_c_ is valid^43^:

| $V_{h}=\frac{V^{*}}{6\ln(l_{1}/e)}\theta_{D}^{3}$ | (S1), |
| --- | --- |

where *V** is characteristic velocity, *l*_1_ is the size of the marginal swelling around the hole (Figure S3 **C**). This equation indicates that the characteristic velocity of a liquid can be determined if the spreading velocity of the nucleation-and-growth-type dewetting and the dynamic wetting angle of the liquid on the substrate can both be obtained.

　Our *in-situ* observations succeeded in capturing the nucleation-and-growth-type dewetting dynamics of the unknown water at the water-ice V interface after the compression corresponding to 2.3 GPa effective overpressure (Figures S3 **A** and **B** and SI Video S2). These observations were performed at −10 ±1 °C by using a differential-interference contrast microscope (the conditions for two-phase coexistence of water and ice V are −10 °C and 443 MPa). The frequency and applied peak-to-peak voltage of the square wave to drive crystallization were 250 mHz and 7 V_pp_, respectively. After the crystal growth of ice V by compression (accompanied by the formation of a macroscopically smooth the unknown water-water interface), a hole nucleated in the unknown water thin film on ice V and grew by spreading radially over the ice surface. The observed dynamics were probably due to the formation of a thin layer of the unknown water with a thickness below the critical thickness by the compression. By fitting the time dependence of the radius of the hole, *r*, using a linear function(Figure S3 **D**), the spreading velocity can be measured. The spreading velocities of 6 holes were measured. As a result, the spreading velocity of the unknown water was determined to be 2.23±0.13 μm/sec.

On the other hand, the dynamic wetting angle of the thin layer of the unknown water was estimated by analyzing the shift in the interference fringes in the *in-situ* interferogram (Figure S4) (SI Video S4). Dewetting dynamics of the unknown water was observed by using the interferometer (Figure S2) instead of a differential-interference contrast microscope. Other experimental conditions were the same as those for the observation of the nucleation-and-growth dewetting dynamics. Figure S4 **A** is bright-field image without an interferogram, showing an ice V single crystal before and after compression. The bright-field image after compression shows a wave-like morphology of the unknown water as a result of dewetting. The observations by interferometer were performed for morphology after the dewetting, because the crystal face was well developed and parallel to the roof face of the anvil, which played the role of the reference mirror for the interferometer (Figure S4 **B** inset). As shown in the schematic representation of the optical path in Figure S4 **B** inset, two types of interference fringe can be generated for the cases with or without a thin layer of unknown water, respectively; the optical paths for these are indicated by the pink and orange solid arrows, respectively. The difference between the two optical paths is equal to the thickness of the thin layer of the unknown water. The interference fringes appear as nearly parallel and equally spaced straight lines on the flat interface. Therefore, both optical paths reflected on the bulk water-ice V interface and a bulk water-unknown water interface produced parallel and equally spaced straight-line interference patterns. On the other hand, the interference fringes crossing the boundary between the region with and without an unknown water layer were horizontally shifted at the boundary because of the small difference in the length of the optical paths. The relationship of the thickness of the thin layer and the shift in the interference fringe from the straight lines of interference fringes on the reference surface (the bulk water-ice V interface), equal to Δ*l* in our experimental situation, can be expressed as follows^44^:

| $e=\frac{\lambda}{2n_{w}}\cdot\frac{\Delta l}{l_{0}}$ | (S2), |
| --- | --- |

where  is the wavelength of the light of the interferometer ( = 632.8 nm), *n_w_* is refractive index of water, *l*_0_ is the interval between the straight lines of the interference fringes on the reference interface. Therefore, an analysis of the horizontal shift of the interference fringes allowed us to estimate the thickness of the thin layer of the unknown water. Figure S4 **B** shows the *in-situ* interferograms in which a horizontal shift of the interference fringes was successfully observed. Figure S4 **C** is a magnified image of the interferogram. The horizontal shift of interference fringes can be observed at the boundary between the region with and without thin film of the unknown water. From these observations, the thickness of the thin layer was estimated to be about 88 nm by using the value of *n_w_* at −10 °C and 443 MPa estimated by the method discussed in Text S2. The estimated value of *n_w_* was 1.39. The interferogram indicates that the distance required to cross the boundary between the region with the thin layer to that without such a layer, Δ*L*, is about 9 μm, suggesting that there is a slope with a height increment of 88 nm over the 9 μm distance. Therefore, the wetting angle of the thin layer of the unknown water can be estimated to be about 0.56° (0.009 rad) by assuming that tan*θ* is equal to $e/\Delta L$ because $\Delta L$ is significantly larger than $e$. Because Figure S3 **B** suggests that the value of *l*_1_ is about 10 μm, the characteristic velocity of the unknown water can be estimated to be about 90 m/sec from eq. S1.

Supplementary Information Text S2

**Estimation of the refractive index of bulk water at −10^o^C and 443 MPa.**

To obtain the value of the refractive index of bulk water at −10 °C and 443 MPa, we referred to the dataset for the temperature and pressure dependence of the refractive index provided by Cho *et al.*^45^ and Martín-Sánchez *et al.*^46^, respectively. The temperature dependence at 1 atm is based on the Lorentz-Lorentz equation using a two-state formalism of water for its specific refraction^45^, and this agrees with experimentally based values from the equation of Tilton and Taylor to within 1 × 10^−5^ or better for temperature from −10 to +70 °C^47^. We first obtained the refractive index of bulk water at −10 °C and 1 atm by extrapolating the temperature dependence for the range −5 to +5 °C for  = 501.568 nm (as provided by Tilton and Taylor^47^) to −10 °C. The refractive index at −10 °C and 1 atm was calculated to be 1.33745 as shown by our previous study^20^. This value was then applied to the Murnaghan-type equations showing the pressure dependency provided by Martín−Sánchez *et al.*^46^:

| $n= n_{0}\left( \frac{P\alpha}{\beta}+1 \right)^{1/\alpha}$ | (S3), |
| --- | --- |

where *n*_0_ is refractive index at 1 atm, *P* is pressure in GPa, α and β are empirical parameters and those values are 26 and 6, respectively, for water at room temperature in the range from 0 to 1.8 GPa. By substituting 1.33745, which is the refractive index at −10^o^C and 1 atm, to eq. S3 as *n*_0_, the value of about 1.39 can be obtained as the refractive index of water at −10^o^C and 443 MPa.

Supplementary Information Text S3

**The density of the unknown water at the interface between water and ice V.**

Here, we qualitatively discuss whether the density of the unknown water is higher than that of the surrounding bulk water based on Young’s equation given by^48^:

| $\gamma_{IW}=\gamma_{IL}+\gamma_{LW}cos\theta$ | (S4), |
| --- | --- |

where $\gamma_{IW}$, $\gamma_{IL}$, and $\gamma_{LW}$ are the ice V-bulk water, ice V-newly discovered unknown water, and the unknown water-bulk water interfacial free energies, respectively. In single component system of water, balances among interfacial free energies should be strongly governed by the structures of phases rather than varieties of intermolecular interaction in contrast to those in multi component systems. This is because the factor to vary or modulate interfacial free energies in single component system should be limited to the structures of the phases unlike that in multi component systems. Since the structures of phases in singe component system are directly related to densities of the phases, the balances among interfacial free energies in single component system should directly reflect the relationship in densities of the phases. Therefore, Young’s equation enables us to estimate whether the density of the unknown water is higher than that of the surrounding bulk water. From Young’s equation, the constraint *θ* < 90° leads to $\gamma_{IW}$ > $\gamma_{IL}$, implying that the structure of the liquid thin layer is more similar to that of ice V with a higher density than that of bulk water compared with that of bulk water. Since the wetting angle of the unknown water was estimated to be significantly smaller than 90^o^ in the Text S1 (below 1^o^), the density of the unknown water at the water-ice V interface is probably higher than that of bulk water.

Supplementary Information Text S4

**Measurement of the characteristic length of spinodal decomposition-like dynamics of the unknown water.**

The wavelength of spinodal wave is generally known as characteristic length. To investigate the similarity of spinodal decomposition-like dynamics of the unknown water with spinodal decomposition in LLPS of binary liquid mixture, we measured the wavelength of the spinodal-like dynamics of the unknown water as the characteristic length. Figure S5 **A** shows an example of the time-lapse micrographs showing spinodal-like dynamics of the unknown water the wavelength of which were measured (SI Video S5). The wavelengths were measured by plotting brightness line profiles of the spinodal-like wave (Figure S5 **B**). Time evolution of the characteristic length of spinodal-like wave was investigated for the spinodal-like dynamics driven by the compression corresponding to 2.3 GPa effective overpressure attained by the voltage of 7 V_pp_ outputted from the function generator connected to the piezo actuator in d-SAC. In addition, the dependencies of the characteristic wavelength on the effective overpressures were investigated using the micrographs taken 0.2 and 1.7 sec after the compression in each applied overpressure.


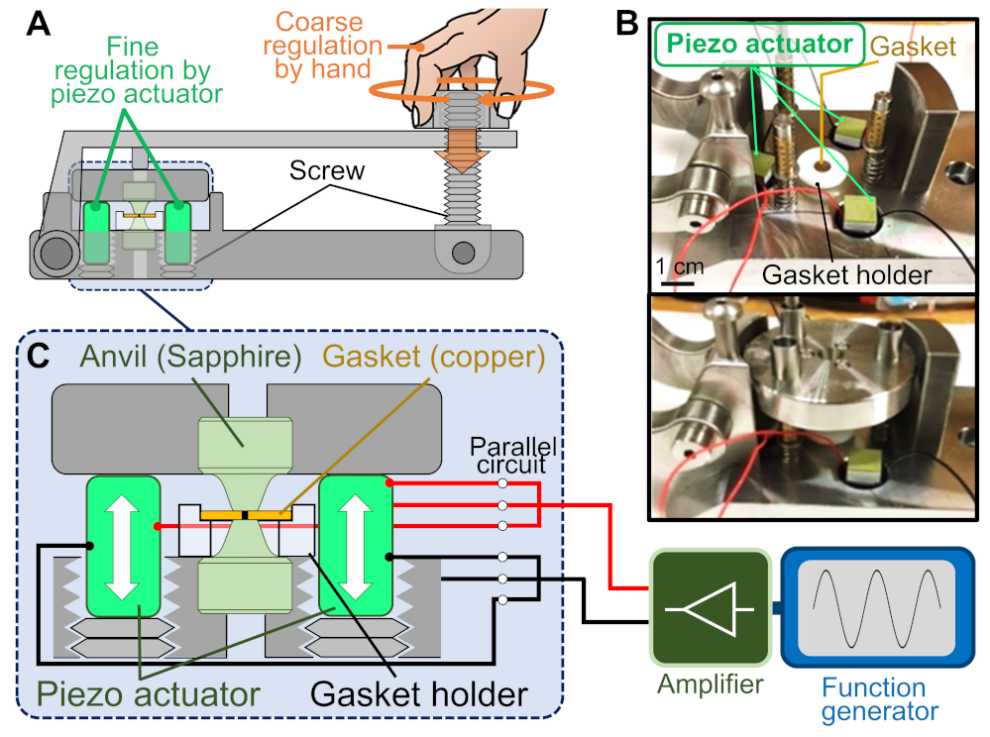


Figure S1. Electrically regulated anvil cell with piezo actuator, dynamic sapphire anvil cell (d-SAC) (Reprinted with permission from ref. 20. Copyright 2022 American Chemical Society.). (A) Schematic overview of the d-SAC. The pressure can be roughly adjusted by manually tightening the screw. The preparation of a single crystal of ice V and presetting of the pressure before the observations were carried out by this rough regulation. Subsequent fine regulation of the pressure for the observations was conducted by using the three electrically driven piezo actuators. (B) Bird’s-eye view of the metal jig supporting the lower anvil (upper) and view after setting the jig supporting the upper anvil (lower). The lower jig has three holes to contain the piezo actuators at intervals of 120° with the anvil at the centre. (C) The magnified schematic of the region highlighted in blue in schematic A. The three piezo actuators are connected through a parallel circuit to the function generator as the electrical source and an amplifier as the piezo controller.


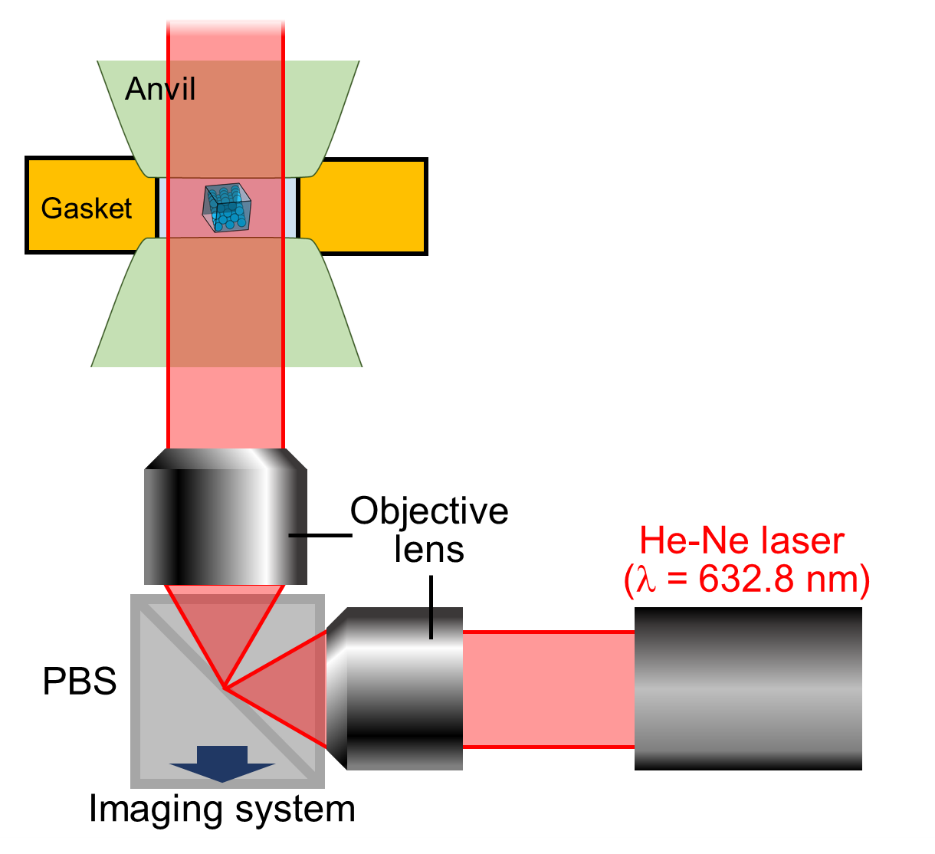


Figure S2. Experimental setup of the interferometer and *in-situ* observation of the unknown water at the water-growing ice V interface with the interferometer (Reprinted with permission from ref.20. Copyright 2022 American Chemical Society).


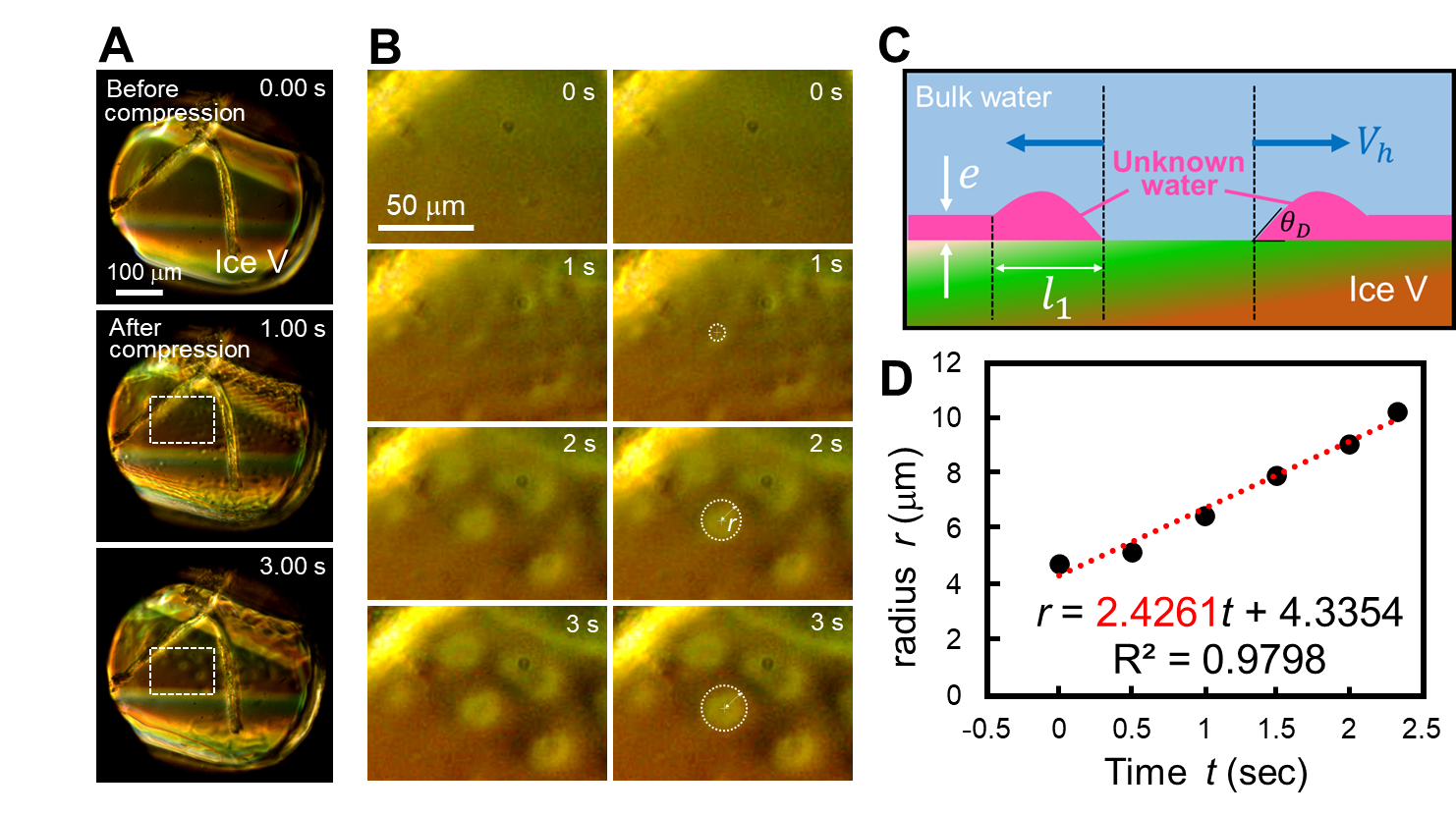


**Figure S3. Analysis of nucleation-and-growth-type dewetting dynamics of the unknown water at the water-ice V interface.** (**A**) Time-lapse *in-situ* optical micrographs showing the nucleation-and-growth-type dewetting dynamics of the thin film of the unknown water at water-ice V interface induced by compression corresponding to 2.3 GPa effective overpressure. (**B**) Magnified time-lapse micrographs without (left) and with (right) eye guides showing the nucleation-and-growth-type dewetting dynamics of the unknown water, which took place in the region indicated by the dashed square in **A** after pressurization. The white dotted circle of radius *r*, denoted by the white arrow, indicates the hole of the thin film of the unknown water resulting from dewetting. Although the hole was not completely circular in the later stages of the dynamics, for the sake of simplicity we measured its radius as that of a complete circle, regarding the longest distance from the centre of the hole to the front line of the dewetting as the radius of the circle. (**C**) A schematic showing a simplified model of the cross-section of the thin layer of the unknown water and ice V when nucleation-and-growth-dewetting dynamics occur. (**D**) Dependence of the radius of the hole in the thin film of the unknown water on time. The black points indicate the measured radius at each time. the red dotted line is the result of linear fitting. The equations in the graph indicate the fitting function and the coefficient of determination. See also SI Video S2.


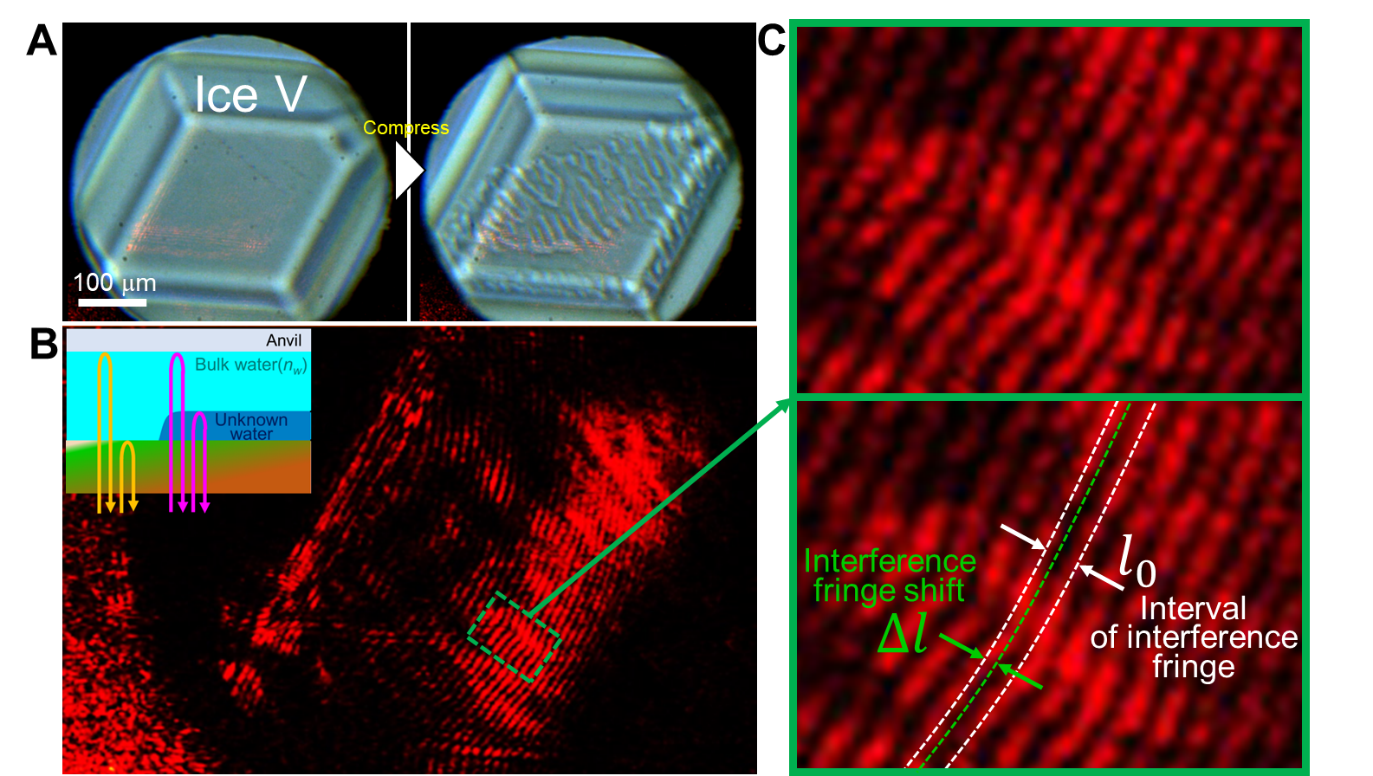


**Figure S4.*****In-situ* interferogram of the thin layer of the unknown water at the water-ice V interface.** (**A**) Bright-field time-lapse micrographs showing the water−ice V interface at which the interferometric observations were performed. The left-hand micrograph is before compression and the right-hand one is after compression. The compression resulted in dewetting of the thin film of the unknown water. (**B**) Interferograms showing horizontal shift in the interference fringes. The interferogram shows the horizontal shift in the interference fringes reflecting the morphology of the interface between water and the unknown water. The inset shows a schematic illustration showing the two possible optical paths that could give rise to the observed interference fringes, indicated by orange and pink arrows, respectively. The optical path indicated by the orange arrows involves interference between light reflected by the bulk water-anvil interface and that reflected by the bulk water-ice V interface. The path indicated by the pink arrows shows the interference between the light reflected by the bulk water-anvil interface and that reflected by the bulk water-the unknown water interface. These optical paths practically correspond to that of a Michelson-type interferometer, with the bulk water/anvil interface acting as a reference mirror, giving interference fringes with parallel and equally spaced lines. The small difference in height between the interface with and without the thin layer of the unknown water (*e*) causes a shift in the interference fringes when one crosses the boundary from the region with the thin film of the unknown water to that without such a film. (**C**) Magnified interferograms of the region indicated by the green dotted square in the lower-right-hand micrograph in **B**. A horizontal shift in the interference fringe, denoted by Δ*l*, can be observed at the boundary between the region with and without thin film of the unknown water, as indicated by the green dotted line. See also SI Video S4.


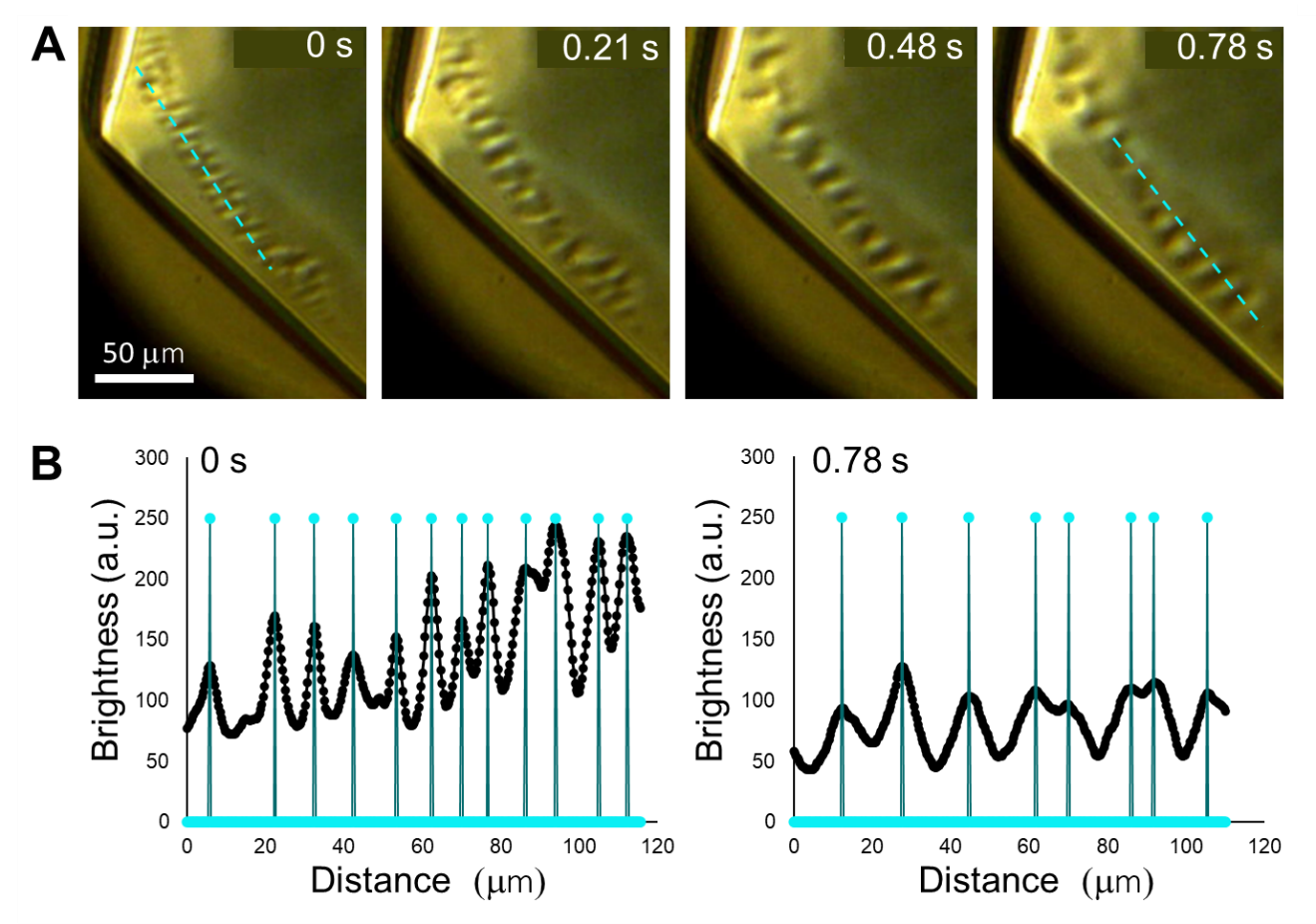
Figure S5. Measurement of the characteristic length of the spinodal-like dynamics of the unknown water. (A) Time-lapse micrographs showing the *in-situ* observation of the spinodal-like dynamics at the interface between water and ice V grown by compression of 2.3 GPa effective overpressure driven by applying the voltage of 7 Vpp outputted from the function generator in the d-SAC. See also SI Video S5. The dashed cyan line indicates the examples of the region where line profiles of brightness was measured as shown in B. (B) Examples of the brightness line profiles taken from the dashed cyan lines indicated micrographs in A. Black profiles indicate the brightness profile. Cyan dots and lines indicate the peak position of the brightness line profiles.

Legends for SI Videos S1-S5

SI Video S1 (separate file). Spinodal-like Generation of Unknown Water at the Interface between Water and Ice V Grown by Compression. The video Corresponds to Figure 2

SI Video S2 (separate file). Nucleation-and-Growth Type Dewetting Dynamics of the Thin Film of the Unknown Water. The video Corresponds to Figure S3.

SI Video S3 (separate file). Spinodal-like Dewetting Dynamics with Anisotropy of the Thin Film of Unknown Water. The video Corresponds to Figure 4.

SI Video S4 (separate file). *In-Situ* Micro-Interferogram of the Dewetting Dynamics of the Thin Film of Unknown Water. The video Corresponds to Figure S4.

SI Video S5 (separate file). *In-Situ* Observation of Spinodal-like Wave of the Unknown Water for the Investigation of Time-Evolution of the Characteristic Length. The video Corresponds to Figure S5.

SI References

43. de Gennes, P. G., Wyart, F. B. & Quéré, D. *Capillarity and Wetting Phenomena: Drops, Bubbles, Pearls, Waves*, Springer: Switzerland, 2003.

44. Nagashima, K., Maurais, J., Murata, K., Furukawa, Y., Ayotte, P. & Sazaki, G. Appearance and disappearance of quasi-liquid layers on ice crystals in the presence of nitric acid gas. *Crystals* **10**, 72 (2020).

45. Cho, C. H. Urquidi, J. Mixture model description of the T-P dependence of the refractive index of water. *J. Chem. Phys.* **114**, 3159–3162 (2001).

46. Martín-Sanchez, C., Gonzalez-Rubio, G., Mulvaney, P., Guerrero-Martínez, A., Liz-Marzan L. M. & Rodríguez, F. Monodisperse Gold Nanorods for High-Pressure Refractive Index Sensing. *J. Phys. Chem. Lett.* **10**, 1587–1593 (2019).

47. Tilton, L. W. & Taylor, J. K. Refractive index and dispersion of distilled water for visible radiation, at temperatures 0 to 60 °C. *J. Res. Natl. Bur. Stand. (U. S.)* **20**, 419–477 (1938).

48. Young, T. An essay on the cohesion of fluids. *Phil. Trans. R. Soc. London* **95**, 65–87 (1805).
